# Genotyping-by-Sequencing Based Genetic Mapping Identified Major and Consistent Genomic Regions for Productivity and Quality Traits in Peanut

**DOI:** 10.3389/fpls.2021.668020

**Published:** 2021-09-23

**Authors:** Mangesh P. Jadhav, Sunil S. Gangurde, Anil A. Hake, Arati Yadawad, Supriya S. Mahadevaiah, Santosh K. Pattanashetti, M. V. Channabyre Gowda, Kenta Shirasawa, Rajeev K. Varshney, Manish K. Pandey, Ramesh S. Bhat

**Affiliations:** ^1^Department of Biotechnology, University of Agricultural Sciences, Dharwad, India; ^2^Center of Excellence in Genomics & Systems Biology (CEGSB), International Crops Research Institute for the Semi-Arid Tropics (ICRISAT), Hyderabad, India; ^3^Department of Genetics and Plant Breeding, University of Agricultural Sciences, Dharwad, India; ^4^Department of Frontier Research and Development, Kazusa DNA Research Institute, Chiba, Japan

**Keywords:** peanut, productivity and quality traits, GBS, transposable elements, SSRs, main and epistatic QTL, QTL validation

## Abstract

With an objective of identifying the genomic regions for productivity and quality traits in peanut, a recombinant inbred line (RIL) population developed from an elite variety, TMV 2 and its ethyl methane sulfonate (EMS)-derived mutant was phenotyped over six seasons and genotyped with genotyping-by-sequencing (GBS), *Arachis hypogaea* transposable element (AhTE) and simple sequence repeats (SSR) markers. The genetic map with 700 markers spanning 2,438.1 cM was employed for quantitative trait loci (QTL) analysis which identified a total of 47 main-effect QTLs for the productivity and oil quality traits with the phenotypic variance explained (PVE) of 10–52% over the seasons. A common QTL region (46.7–50.1 cM) on Ah02 was identified for the multiple traits, such as a number of pods per plant (NPPP), pod weight per plant (PWPP), shelling percentage (SP), and test weight (TW). Similarly, a QTL (7.1–18.0 cM) on Ah16 was identified for both SP and protein content (PC). Epistatic QTL (epiQTL) analysis revealed intra- and inter-chromosomal interactions for the main-effect QTLs and other genomic regions governing these productivity traits. The markers identified by a single marker analysis (SMA) mapped to the QTL regions for most of the traits. Among the five potential candidate genes identified for PC, SP and oil quality, two genes (*Arahy.7A57YA* and *Arahy.CH9B83*) were affected by *AhMITE1* transposition, and three genes (*Arahy.J5SZ1I, Arahy.MZJT69*, and *Arahy.X7PJ8H*) involved functional single nucleotide polymorphisms (SNPs). With major and consistent effects, the genomic regions, candidate genes, and the associated markers identified in this study would provide an opportunity for gene cloning and genomics-assisted breeding for increasing the productivity and enhancing the quality of peanut.

## Introduction

Peanut or groundnut (*Arachis hypogaea* L. 2*n* = 4x = 40) is an important oilseed, legume food, and fodder crop, which, in 2019, was cultivated globally on an area of 29.5 million ha with a production of 48.7 million tons and a productivity of 1,647 kg/ha (http://www.fao.org/faostat/en/#data/QC/visualize). Globally, over half of the peanut produce goes for oil extraction while the remaining is consumed as raw and processed food. In India, over 80% of the produce was used for oil extraction in the past. But, now, it has reduced to <50% (Sharma, 2017), indicating a shift in the use of peanut in multiple food preparations. Apart from being rich in oil, proteins, fibers, polyphenols, antioxidants, vitamins, and minerals, the peanut is an excellent source of compounds, such as resveratrol, phenolic acids, flavonoids, and phytosterols, co-enzyme Q10, and amino acids (all 20, with the highest content of arginine). Peanut forms a major food component in fighting malnutrition in the form of Ready-to-use Therapeutic Food (RUTF) in Africa and Asia. With these nutrient profiles, peanut is being considered a functional food (Arya et al., 2016). Thus, peanut has gained the status of “poor person's almond” over the years. However, kernel features and nutritional qualities need to be considered while attempting to increase peanut productivity along with tolerance to the biotic and abiotic stresses.

The last decade has been transformational for peanut stakeholders globally because of tremendous developments in the availability of substantial genomic resources and optimization of multiple modern breeding approaches, such as marker-assisted selection (MAS), genomic selection, and rapid generation advancements (as shown in Pandey et al., 2020). The availability of high-quality reference genomes for diploid subgenomes (Bertioli et al., 2016; Chen et al., 2016; Lu et al., 2018), primitive tetraploid (Yin et al., 2018) as well as the subspecies of the cultivated tetraploid peanut (Bertioli et al., 2019; Chen et al., 2019; Zhuang et al., 2019), high density genotyping assay with 58K single nucleotide polymorphisms (SNPs) (Pandey et al., 2017), genotyping-by-sequencing (GBS) (Dodia et al., 2019; Wang et al., 2019, 2021; Zhou et al., 2021), and other reduced-representation sequencing (Zhao et al., 2016; Shirasawa et al., 2018; Luo et al., 2021) based genotyping in peanut provided a strong platform for precise trait mapping, gene discovery, and marker development for use in breeding (Han et al., 2018; Wang et al., 2019). With the availability of trait-specific markers, peanut has already demonstrated the application of marker-assisted breeding by developing several new varieties with improved disease resistance and oil quality (as shown in Pandey et al., 2020). However, the challenge still prevails for molecular breeding to improve the productivity traits that show complex genetic inheritance. Therefore, such traits need multi-environment phenotyping and dense genotyping data for performing high-resolution genetic mapping and the precise detection of genetic factors with direct and epistatic effects over the seasons.

The recombinant inbred line (RIL) population (Pattanashetti, 2005) derived from an elite peanut variety TMV 2 and its ethyl methane sulfonate (EMS)-induced mutant TMV 2-NLM (Prasad et al., 1984) allowed subtracting a large portion of the genome common between the parents, thereby favoring successful trait mapping as demonstrated with 105 AhTE markers in our previous effort (Hake et al., 2017). Therefore, this study aimed to enrich the linkage map with GBS-based SNP markers along with AhTE and simple sequence repeat (SSR) markers and generating the phenotypic data over six seasons to detect the genomic regions with main and epistatic effects in addition to identifying a few co-segregating genes.

## Materials and Methods

### Plant Material

We used a RIL population developed (Pattanashetti, 2005) from the cross between TMV 2, an elite variety of peanut and its EMS-mutagenized derivative TMV 2-NLM (Prasad et al., 1984). TMV 2 is a Spanish bunch cultivar known for its uniform pods and kernels, kernel taste, and wide adaptability (Rathnakumar et al., 2013), but low in OLE (42.08%). TMV 2-NLM is a semi-spreading cultivar with bold kernels, low yield, and moderate content of oleic acid (53.73%) (Prasad et al., 1984). The phenotypic and genotypic differences between TMV 2 and TMV 2-NLM have been previously reported by Hake et al. (2017).

### Phenotyping of the Mapping Population and Statistical Analysis

**Figure 1:**
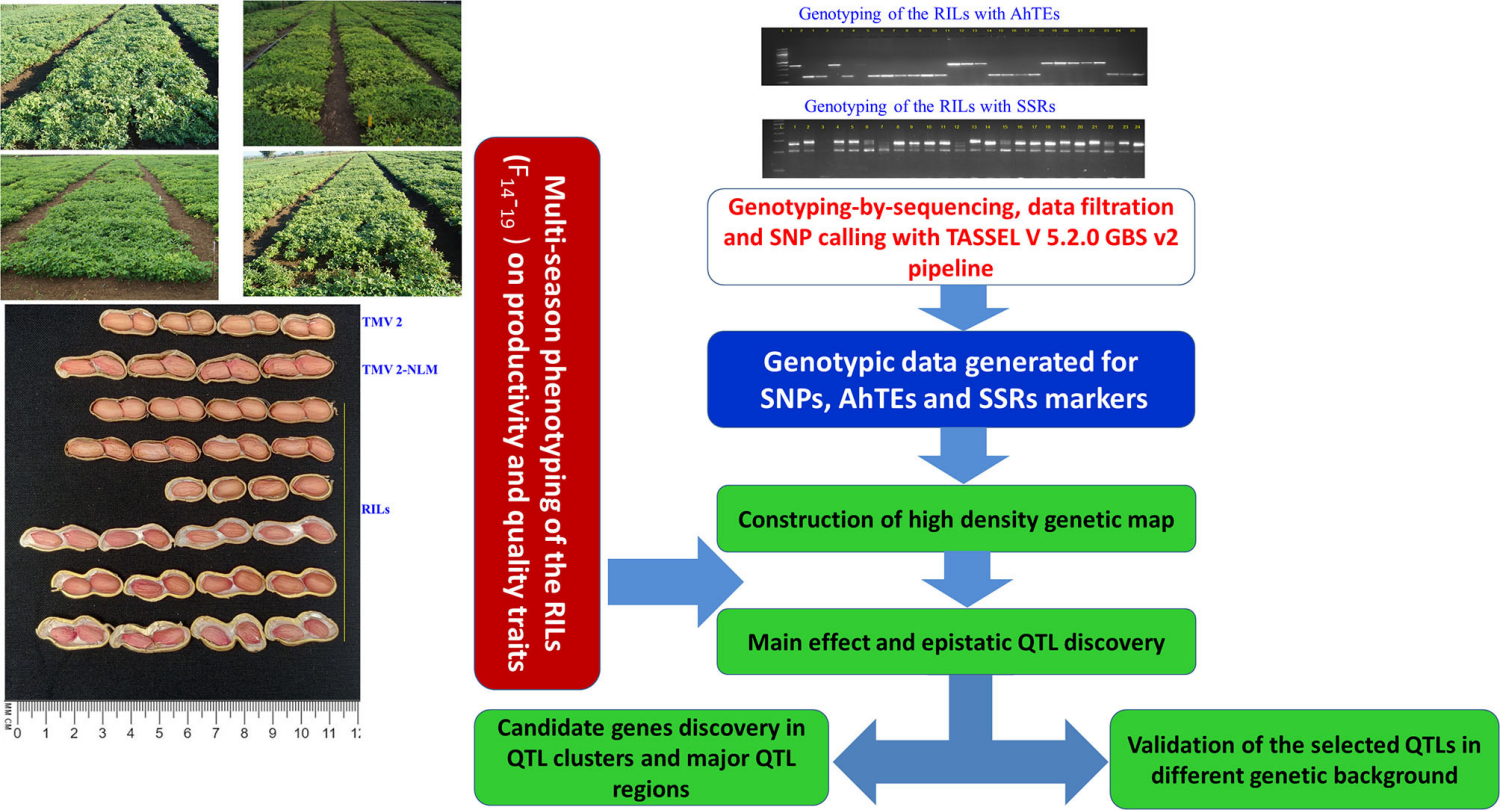
Flow chart of genotyping, high density genetic map construction, multi-season phenotyping, identification of genomic regions for productivity, and quality traits and their validation.

F_14−19_ generations of the 432 RILs together with the parents were grown during the six seasons, namely, rainy 2014 (S1), rainy 2015 (S2), rainy 2016 (S3), rainy 2017 (S4), rainy 2018 (S5), and post-rainy 2018 (S6) at IABT garden (E115) of Main Agricultural Research Station, University of Agricultural Sciences, Dharwad, India (Figure 1). During each season, the RILs were grown in two replications with a spacing of 30 × 10 cm with recommended agronomic practices. The observations were recorded on productivity traits, such as the number of pods per plant (NPPP), pod weight per plant (PWPP), shelling percentage (SP), and test weight (TW), and on quality traits, such as protein content (PC), oil content (OIL), OLE, linoleic acid content (LIN), and oleic to linoleic acid ratio (O/L). The quality parameters were estimated using the near-infrared reflectance spectroscopy (NIRS) (Model XDS RCA, FOSS Analytical AB, Sweden, Denmark) at ICRISAT, Patancheru, India.

An ANOVA was performed for each trait observed during each season to test the significant differences among the RILs. A pooled analysis of variance was performed for all the traits across the seasons allowing G × E interactions. Phenotypic coefficient of variation (PCV), genotypic coefficient of variation (GCV), and broad sense heritability (h^2^b.s) were estimated using the plant breeding package Windostat ver. 8.5 (Indostat Services, Hyderabad, India, https://www.indostat.org/agriculture.html). Pearson's correlation coefficients (*r*) among the different traits were estimated over the seasons using the 16th version of SPSS (SPSS Inc., Chicago, IL, USA).

### DNA Extraction and Genotyping With AhTE and SSR Markers

DNA was isolated from the young leaves of each RIL and the parents following the modified cetyltrimethyl-ammonium bromide (CTAB) method as described by Cuc et al. (2008). DNA quality of each sample was checked on 0.8% agarose gel. Furthermore, DNA quantification was done using Nano-Drop (UV technologies, Wilmington, DE, USA), and DNA concentration was normalized to ~5–10 ng/μl for genotyping the parents and the RIL population using AhTE and SSR markers. In total, 343 AhTE markers (Gayathri et al., 2018) and 91 SSR markers (as shown in Pandey et al., 2012) were screened for parental polymorphism between TMV 2 and TMV 2-NLM. Subsequently, the markers polymorphic between the parents were identified and used to genotype the RILs (Figure 1). PCR and separation of the amplicons and scoring of the alleles were performed as described by Kolekar et al. (2016). Genotypic data on 105 AhTE markers generated by Hake et al. (2017) on these RILs were also employed for genetic mapping.

### GBS of the RILs, Sequence Analysis and SNP Calling

Genotyping-by-sequencing was performed for the RILs and their parents as described by Dodia et al. (2019). To perform GBS, 10 ng DNA from each RIL was digested using the restriction endonuclease enzyme *ApeKI* that recognizes the site G/CWCG. The ligation enzyme, *T4* ligase, was used to ligate the digested products with uniquely barcoded adapters. Such digestion and ligation were performed for each RIL, and an equal proportion of the products from each sample was mixed to construct the libraries. These libraries were amplified and purified to remove the excess adapters. They were sequenced on HiSeq 2500 platform (Illumina Inc., San Diego, CA, USA) to generate genome-wide sequence reads.

The sequence reads for the parents and the RILs were obtained as FASTQ files, which were used for SNP discovery using TASSEL version 5.2 (Bradbury et al., 2007) (Supplementary Figure 1). Initially, the perfectly matched barcodes were detected with four bases remnants of the digestion site of the restriction enzyme in the sequencing reads generated for RILs and parental genotypes. Reads were sorted and de-multiplexed using the barcodes. They were trimmed for the first 64 bases starting from the recognition site of the restriction enzyme. Reads containing “N” within the first 64 bases were identified and discarded. Reads passing the quality filtering criteria were mapped onto the reference genome of cultivated peanut *A. hypogaea* (Bertioli et al., 2019) using the Burrows-Wheeler Alignment (BWA) tool (Li and Durbin, 2009). The mapped reads were exported in the form of Sequence Alignment Map (SAM) file. Furthermore, the alignment file was processed for SNP calling using SNP caller plugin implemented in TASSEL version 5.2.0 GBS v2 pipeline as per the standard instruction (https://bitbucket.org/tasseladmin/tassel-5-source/wiki/Tassel5GBSv2Pipeline). The RILs with <85 Mb data were not processed for further analysis to avoid false-positives. The SNPs with more than 50% missing data and minor allele frequency (MAF) of ≤ 0.3 were filtered out to avoid the noise during genetic map construction. The SNPs with <50% missing data for the RILs were imputed using Beagle version 5.2 (Browning et al., 2018) algorithm. Furthermore, filtering was performed to check the percentage heterozygosity and polymorphic SNPs between the parents (Supplementary Figure 1).

### Genetic Map Construction

High-quality SNPs obtained after filtering were further considered for genetic analysis. A chi-square (χ^2^) test was applied on polymorphic AhTE, SSR, and SNP markers with a null hypothesis that two alleles from both parents of RIL population at a particular locus segregate in a 1:1 ratio. The markers showing high segregation distortion (χ^2^-test, *P* < 0.001) were filtered out and not considered for the linkage map construction. The genetic map was constructed using JoinMap (version 4.0) (Van Ooijen, 2006) with logarithm of odds (LOD) threshold ranging from 3.0 to 10.0 and a minimum recombination threshold of 45%. Grouping and ordering of the markers were performed using the regression mapping algorithm. Kosambi map function (Kosambi, 1943) was used for genetic map construction, and to convert the recombination frequencies into map distances in centiMorgans (cM). Chromosome-wise marker positions with their respective names were used to draw the final genetic map using MapChart (Voorrips, 2002). The mapped markers were also analyzed for their genic and non-genic location and functional annotation (especially for the SNP and AhTE markers).

### Main-Effect and Epistatic Quantitative Trait Locus Analysis

The main-effect quantitative trait locus (QTL) analysis was carried out using a “composite interval mapping (CIM)” approach (Zeng, 1994) with Model 6 and scanning distance of 1.0 cM between markers and moving window size of 10.0 cM using Windows QTL Cartographer version 2.5 (Wang et al., 2007). A forward–backward stepwise regression method was used to set the marker cofactors for the background selection. The highest peak was considered to locate QTL where the distance between the peak and the QTL was <5.0 cM. Permutation (1,000) test was performed to work out the threshold and identify the significant QTL. The QTLs with >3.0 LOD and phenotypic variance explained (PVE) >10% were considered as major effect QTLs for a particular trait. Those with PVE <10% were considered as minor effect QTLs. Based on the trait name and chromosome number, the QTLs were named, where the first letter “q” indicated the QTL and the abbreviated capital letters indicated the trait followed by chromosome number and the numerical number indicating the serial number of the QTL for a trait. For instance, *qPC-Ah16-1* was the first QTL for PC detected on chromosome Ah16.

Analysis for the epistatic QTL (epiQTL) (Q × Q) was conducted using the function “two-dimensional scanning ICIM-EPI” implemented in inclusive composite interval mapping (ICIM) software version 4.1 (Wang et al., 2014) with 5 cM step and 0.001 probability mapping parameters in stepwise regression. The minimum threshold LOD value for significant epiQTL was set at 3.0.

### Single Marker Analysis

Association of the markers with the productivity and quality traits was tested by SMA using the lm() function (linear regression) of the R program.

### Putative Gene Discovery From Major QTLs/SMA

Putative genes were identified for the major QTLs or QTL clusters. The region between the flanking markers of a particular QTL on the physical map was considered for candidate gene discovery. Where the physical distance between the two flanking markers was more, the marker closer to the peak was selected, and a physical distance of 5 Mb (toward the QTL peak) was searched for the candidate genes. Also, those markers which were identified to be significantly associated with the traits by SMA were checked for their location (genic and non-genic) and effect at PeanutBase (www.peanutbase.com).

### Confirmation of QTLs and Markers

A few selected major QTLs identified in this study were validated using other genotypes (GPBD 4, TG 26, TAG 24, ICGV 86699, ICGV 86855, ICGV 06189, DBG 3, and DBG 4). The markers flanking these QTL were used for genotyping as described above. Co-segregation between the marker and the phenotype was checked using the *t*-test.

## Results

### Phenotypic Variability in the Mapping Population

ANOVA revealed significant differences between the RILs, seasons, and the season × RILs interaction for the productivity and quality traits over the six seasons (Supplementary Table 1). All the traits, except TW and O/L during S6, showed normal distribution based on the kurtosis and skewness (Supplementary Figure 2 and Supplementary Table 2). The RIL population exhibited moderate PCV and GCV for most of the traits (Supplementary Table 2). PC, OIL, and TW showed high broad-sense heritability (${\text{h}}_{\text{bs}}^{2}$), while NPPP, PWPP, SP, OLE, LIN, and O/L revealed low to moderate heritability. Transgressive segregants were observed in both directions for the traits. The correlation analysis showed positive association of NPPP with PWPP (*r* = 0.02–0.62) and SP (0.12^*^-0.18^*^), and a negative association with TW (*r* = −0.06 to −0.14^**^). PC showed positive correlation with NPPP (0.10^*^-0.19^**^), PWPP (0.04-0.11^**^), and SP (0.15^**^-0.24^**^) over the seasons. Similarly, OIL showed a positive correlation with NPPP (0.02-0.17^**^) and PWPP (0.11^*^-0.15^**^) over the seasons. OLE was positively correlated with PWPP (0.03^*^-0.11^*^) and TW (0.09^*^-0.12^**^), however, it was negatively correlated with NPPP (−0.10^*^ to −0.18^**^) and SP (−0.09^*^ to −0.15^**^), PC (−0.32^**^ to −0.34^**^), OIL (−0.03^*^ to −0.29^**^), and LIN (−0.64^**^ to −0.96^**^) (Supplementary Table 3).

### GBS Based High-Density Genetic Map

A total of 1,067.58 million raw reads (100.87 GB) were obtained for the 403 RILs and the two parents. On average, 2.42 million reads (0.23 GB) data were generated for each sample. After filtering, a total of 978.96 million reads (82.9 GB) were mapped onto the tetraploid reference genome of cultivated tetraploid peanut “Tifrunner” (Bertioli et al., 2019). In total, 47,584 raw SNPs (mean read depth of 73.7) were extracted for the downstream analysis. Out of these SNPs, 1,205 polymorphic SNPs were identified between the parental genotypes (TMV 2 and TMV 2-NLM). Further filtering based on missing data and segregation distortion identified 713 SNPs high-quality polymorphic SNPs. The missing data for these SNPs ranging from 0.01 (1%) to 0.489 (48.9%) (Supplementary Table 4).

**Figure 2:**
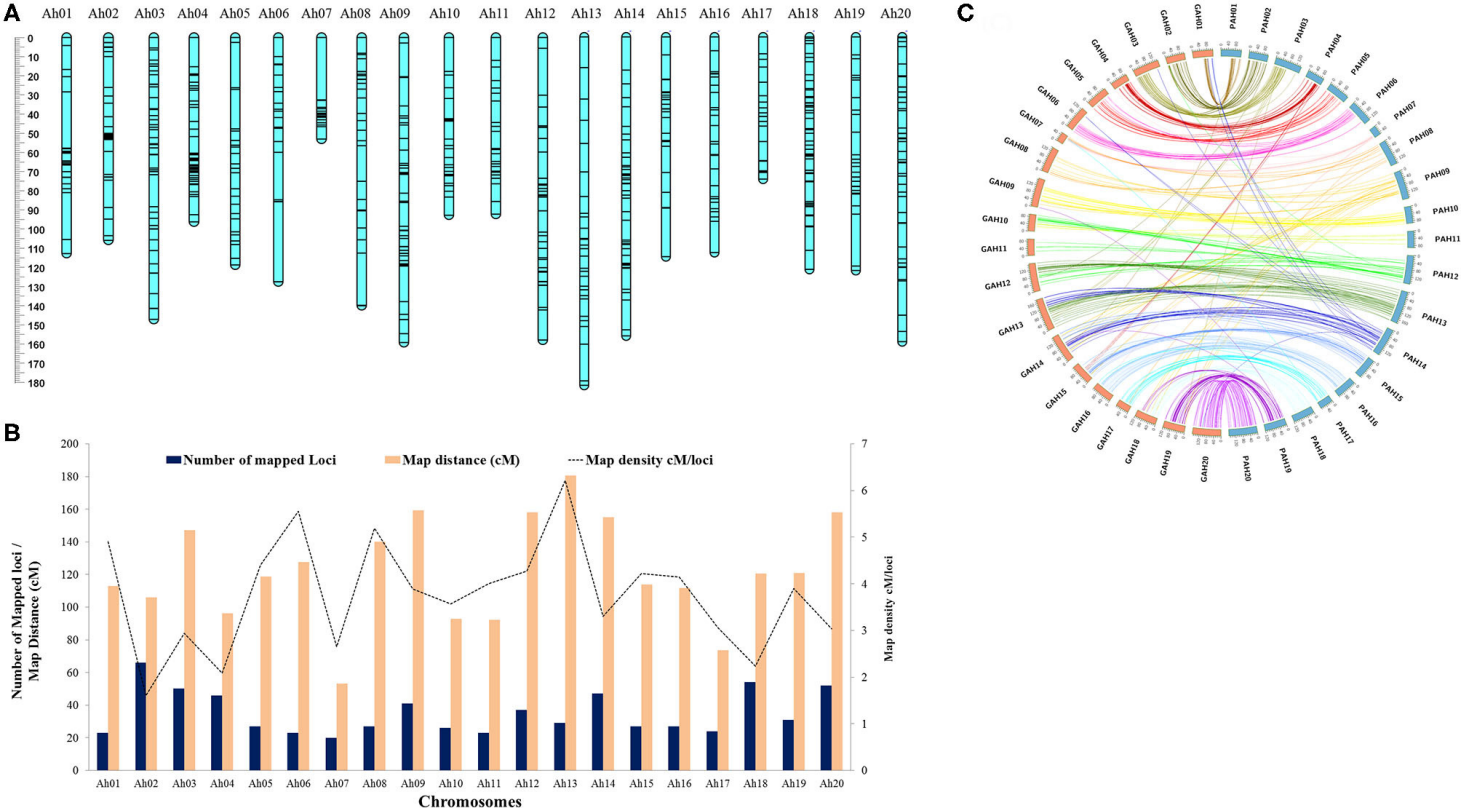
High density genetic map of RIL population of TMV 2 and TMV 2-NLM. **(A)** Map chart of high density genetic map. **(B)** Summary of genetic map with number of mapped loci, map distance (cM), and map density (cM/loci). **(C)** Collinearity of the genetic map with the reference genome (*Arachis hypogaea* L.). Prefix G and P stands for genetic map and physical map, respectively.

Overall genotypic data available for mapping included 865 markers; comprising of 713 SNPs, 143 AhTEs (105 from Hake et al., 2017 and 38 from this study), and 9 SSRs (Supplementary Table 5). The polymorphism percentage for the SNP, AhTE, and SSR markers were 1.49% (713/47,584), 20.08% (143/712), and 9.89% (9/91), respectively. Of these 865 markers, a total of 700 (including 553 SNPs, 136 AhTEs, and 8 SSRs) were mapped to construct a new genetic map spanning a map distance of 2,438.2 cM for this mapping population (Figure 2A and Supplementary Table 6). The genetic map with 20 linkage groups showed a marker density of 3.48 cM/locus. The number of mapped loci ranged from 20 (Ah07 with a density of 2.65 cM/locus) to 66 (Ah02 with a density of 1.60 cM/locus). The length of the chromosomes ranged from 53.06 cM (Ah07) to 180.52 cM (Ah13) (Figure 2B and Supplementary Table 5). Overall, the genetic map showed good marker collinearity with a physical map having a few exceptions (Figure 2C).

### Main-Effect QTL Discovery for Yield and Quality Traits

**Figure 3:**
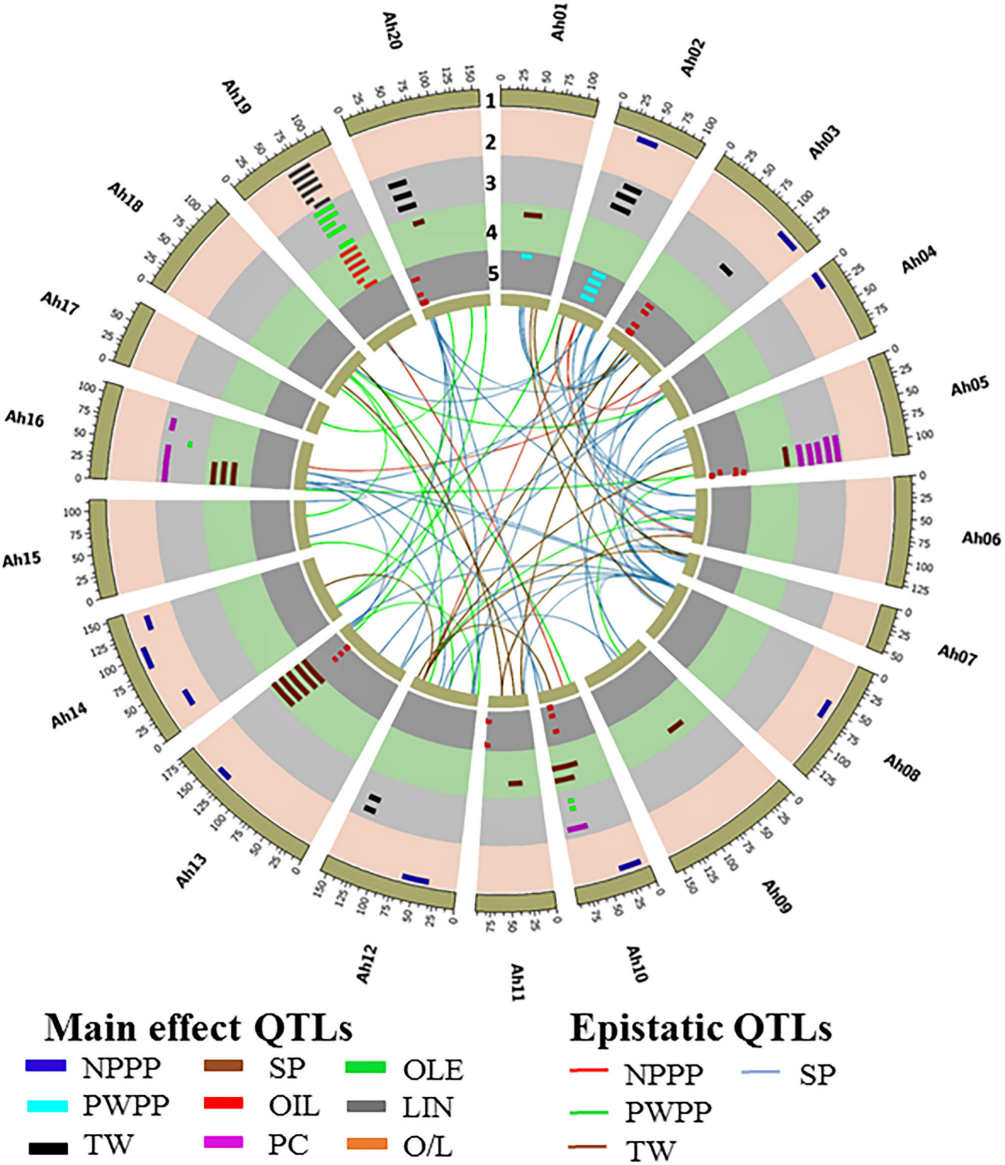
Circos plot illustrating main effect and epistatic (QTL × QTL) QTLs identified for productivity and quality traits in ML population of TMV 2 and TMV 2-NLM of peanut. The tracks from outside to inside indicates (1) 20 chromosomes of tetraploid genome *Arachis hypogaea*, (2) main effect QTLs for number of pods per plant (NPPP) and linoleic acid (IAN) *qNPPP–Ah02-1, qNPPP–Ah04-1, qNPPP–Ah08-1, qNPPP–Ah10-1, qNPPP–Ah12-1, qNPPP–Ah13-1, qNPPP–Ah14-1,qNPPP–Ah14-2, qNPPP–Ah14-3, qLIN–Ah19-1, qLIN–Ah19-2, qLIN–Ah19-3, qLIN–Ah19-4, qLIN–Ah19-5, qLIN–Ah19-6, qLIN–Ah19-7, qLIN–Ah19-8, qLIN–Ah19-9, qLIN–Ah19–10, qLIN–Ah19-11, qLIN–Ah19-12, qLIN–Ah19-13*, (3) main effect QTLs for test weight (TW), protein content (PC), and oleic acid content (OLE) *qTW–Ah02-1, qTW–Ah02-2, qTW–Ah02-3, qTW–Ah02-4, qTW–Ah03-1, qPC–Ah05-1, qPC–Ah05-2, qPC–Ah05-3, qPC–Ah10-1, qOLE–Ah10-1, qTW–Ah12-1, qTW–Ah12-2, qTW–Ah12-3, qTW–Ah12-4, qPC–Ah16-1, qPC–Ah16-2, qPC–Ah16-3, qOLE–Ah16-1, qOLE–Ah19-1, qOLE–Ah19-2, qOLE–Ah19-3*, qOLE–Ah19-4, qOLE–Ah19-5, qOLE–Ah19-6, qOLE–Ah19-7, qOLE–Ah19-8, qOLE–Ah19-9, qOLE–Ah19-10, qOLE–Ah19-11, qOLE–Ah19-12, qTW–Ah20-1, qTW–Ah20-2, qTFV–Ah20-3, (4) main effect QTLs for shelling percentage (SP) and oleic to linoleic ratio (0/L) *qSP–Ah01-1, qSP–Ah02-1, qSP–Ah05-1, q5P–Ah09-1, qSP–Ah10-1, qSP–Ah10-2, qSP–Ah11-1, OP–Ah13-1, OP-A/216-1, qO/L–Ah19-1, qO/L–Ah19-2, qO/L–Ah19-3, qO/L–Ah19-4, q0/L–Ah19-5, qO/L–Ah19-6, qO/L–Ah19-7, qO/L–Ah19-8, qO/L–Ah19-9, qO/L–Ah19-10, qO/L–Ah19-11, qO/L–Ah19-12, qO/L–Ah19-13, qSP–Ah20-1*, (5) main effect QTLs for oil content (OIL) and pod weight per plant (PWPP). *qPWPP–Ah01-1, qPWPP–Ah02-1, qOIL–Ah03-1, qOIL–Ah03-2, qOIL–Ah03-3, qOIL–Ah03-4, qOIL–Ah05-1, qOIL–Ah05-2, qOIL–Ah10-1 qOIL–Ah11-1, qOIL–Ah13-1, qOIL–Ah20-1, qOIL–Ah20-2*. Innermost links connecting between the loci indicates the epistatic QTLs for NPPP, PWPP, TW, and SP.

A QTL analysis was conducted for the productivity (NPPP, PWPP, SP, and TW) and quality (PC, OIL, OLE, LIN, and O/L) traits across six seasons using composite interval mapping at 1,000 permutations. In total, 33 QTLs were identified for the four productivity traits that included nine QTLs for NPPP, two for PWPP, 10 for SP, and 12 for TW (Figure 3 and Supplementary Table 7). Among the nine QTLs for NPPP, three were identified as major QTLs. Among them, the first one (*qNPPP-Ah02-1*) identified on chromosome Ah02 with the LOD score of 9.6 showed 23.6% PVE during S4 season. The second QTL *qNPPP_Ah04-1* detected on Ah04 had a PVE of 22.9% with an LOD score of 3.8 during S1 season. The third QTL *qNPPP_Ah14-3* reported on chromosome Ah14 had a PVE of 17.3% with the LOD score of 7.7 during the S3 season (Figure 3 and Table 1). Among the two QTLs detected for PWPP, the first QTL on Ah02 (*qPWPP-Ah02-1*) was a major QTL with the highest LOD score of 10.6 and PVE of 20.9% (Figure 3 and Table 1), and it was stable over four seasons (S1, S2, S3, and S4). The other QTL on Ah01 (*qPWPP-Ah01-1*) was a minor QTL with a LOD value of 3.3 and a PVE of 5.1%. Of the 10 QTLs for SP, three were major-effect QTLs. Of them, the QTL (*qSP-Ah13-1*) identified on Ah13 had the highest PVE of 52.8% with the LOD score of 35.5 and stability over four seasons (S2, S3, S4, and S5). The second major effect QTL (*qSP-Ah16-1*) on Ah16 identified over three seasons (S1, S2, and S6) with the highest LOD score of 6.6 showed the highest PVE of 19.9% (Figure 3 and Table 1). The third major QTL (*qSP-Ah02-1*) on Ah02 had a PVE of 12.6% with a LOD score of 5.7 was detected only during S3. The remaining seven QTLs identified on Ah01, Ah05, Ah09, Ah10, Ah11, and Ah20 were minor QTLs that appeared only during one or two seasons. The favorable alleles for NPPP and PWPP were contributed by TMV 2-NLM, while TMV 2 contributed the favorable allele at two major QTLs for SP. No major-effect QTL was detected for TW; however, 12 minor-effect QTLs were identified with the highest PVE of 8.5% (*qTW-Ah02-4*).

**Table 1:** Major main effect quantitative trait loci (QTLs) identified for productivity and quality traits across seasons in the recombinant inbred line (RIL) population of TMV 2 and TMV 2-NLM of peanut.

**Trait/QTL**	**Chromosome**	**Peak position (cM)**	**Confidence interval**	**Flanking markers**	**LOD**	**PVE (%)**	**Additive effect**	**Stability across season(s)**
**Number of pods per plant (NPPP)**								
*qNPPP-Ah02-1*	Ah02	48.7	46.7–50.1	Ah02_100281747–Ah02_1558084	9.6	23.6	2.1	S4
*qNPPP-Ah04-1*	Ah04	11.0	0–15.4	AhTE0087–TC11H06	3.8	22.9	2.8	S1
*qNPPP-Ah14-3*	Ah14	133.5	132.5–136.3	Ah11_1177069–Ah14_4643565	7.7	17.3	2.5	S3
**Pod weight per plant (PWPP)**								
*qPWPP-Ah02-1*	Ah02	48.7	46.7–50.1	Ah02_100281747–Ah02_1558084	4–10.6	7.4–20.9	2–7.1	S1, S2, S3, S4
**Shelling percentage (SP)**								
*qSP-Ah02-1*	Ah02	48.7	46.7–50.1	Ah02_100281747–Ah02_1558084	5.7	12.6	6.7	S3
*qSP-Ah13-1*	Ah13	163.3	159.3–178.3	Ah13_80163117–Ah13_50074616	3.2–35.5	16.3–52.8	−6.6 to −1.2	S2, S3, S4, S5
*qSP-Ah16-1*	Ah16	13.1	7.1–18	AhTE0060–Ah16_77480103	4.1–6.6	10.5–19.9	−4.4 to −1.4	S1, S2, S6
**Protein content (PC)**								
*qPC-Ah16-1*	Ah16	3.0	0.0–7.1	AhTE0242–AhTE0060	7.6–15.3	7.9–13.3	0.8–1.3	S1, S3, S4, S5
*qPC-Ah16-2*	Ah16	12.1	7.1–18	AhTE0060–Ah16_77480103	6.9–13.5	9.7–13.3	1.0–1.3	S1, S3, S4, S5
**Oil content (OIL)**								
*qOIL-Ah03-3*	Ah03	37.4	37.4–37.5	Ah03_142744376–AhTE1144	3.3–9.5	6.2–13.7	0.8–1.2	S1, S2, S4, S5
*qOIL-Ah05-1*	Ah05	111.2	108.2–115.3	Ah05_115061124–AhTE0470	3.8–4.6	9.1–10.7	0.6–0.6	S2, S4
**Oleic acid content (OLE)**								
*qOLE-Ah19-1, qOLE-Ah19-2, qOLE-Ah19-3, qOLE-Ah19-4, qOLE-Ah19-5, qOLE-Ah19-6, qOLE-Ah19-7, qOLE-Ah19-8, qOLE-Ah19-9, qOLE-Ah19-10, qOLE-Ah19-11, qOLE-Ah19-12*	Ah19	78.6^*^	61.1–87.6	Ah19_155127299–Ah19_155179303^**^	5.1–22.2	5.5–21.3	−0.1 to −0.28	S1, S2, S3, S4, S5, S6
**Linoleic acid content (LIN)**								
*qLIN-Ah19-1, qLIN-Ah19-2, qLIN-Ah19-3, qLIN-Ah19-4, qLIN-Ah19-5, qLIN-Ah19-6, qLIN-Ah19-7, qLIN-Ah19-8, qLIN-Ah19-9, qLIN-Ah19-10, qLIN-Ah19-11, qLIN-Ah19-12, qLIN-Ah19-13*	Ah19	75.8^*^	61.1–87.6	Ah19_155127299–Ah19_155179303^**^	6.3–18.3	7.0–17.1	0.9–2.2	S1, S2, S3, S4, S5, S6
**Oleic linoleic ratio (O/L)**								
*qO/L-Ah19-3, qO/L-Ah19-4, qO/L-Ah19-5, qO/L-Ah19-6, qO/L-Ah19-7, qO/L-Ah19-8, qO/L-Ah19-9, qO/L-Ah19-10, qO/L-Ah19-11, qO/L-Ah19-12, qO/L-Ah19-13*	Ah19	78.6^*^	61.1–87.6	Ah19_155127299–Ah19_155179303^**^	6.5–19.5	6–18.4	−0.2 to −0.1	S1, S2, S3, S4, S5, S6

* *Peak value of QTL with highest*.

** *Right flanking marker of first QTL and left flanking marker of last QTL; LOD, Logarithm of odds; PVE, Phenotypic variance explained; S1, Rainy 2014; S2, Rainy 2015; S3, Rainy 2016; S4, Rainy 2017; S5, Rainy 2018 and S6, Post-rainy 2018–19*.

For OIL, 11 QTLs were identified, of which two were major, and located on chromosome Ah03 (*qOIL-Ah03-3*) and Ah05 (*qOIL-Ah05-1*). *qOIL-Ah03-3* was detected over four (S1, S2, S4, and S5) seasons with the highest LOD score of 9.5 and PVE of 13.7%. While *qOIL-Ah05-1* was detected over the two seasons (S2 and S4) with the highest LOD score of 4.6 and PVE of 10.7%. The favorable alleles at both these QTLs were contributed by TMV 2-NLM (Figure 3 and Table 1).

A total of 47 QTLs were identified for quality traits (PC, OLE, LIN, and O/L) (Figure 3 and Supplementary Table 7). For PC, out of seven QTLs, two QTLs on Ah16 were major and stable across four seasons. Of them, *qPC-Ah16-1* had the highest PVE of 13.3% with a LOD score of 15.3, and *qPC-Ah16-2* had the highest PVE of 13.3% with a LOD score of 13.5. The favorable alleles for both these QTLs were contributed by TMV 2-NLM (Figure 3 and Table 1). A total of 14 QTLs were identified for OLE along with 13 QTLs each for LIN and O/L. Of them, 12 QTLs for OLE were major and stable with the highest PVE of 21.3%. The remaining two minor QTLs were mapped on Ah10 and Ah16 (Figure 3, Table 1, and Supplementary Table 7). For LIN, all the 13 QTLs were major and stable with the highest PVE of 17.1% (Figure 3, Table 1, and Supplementary Table 7). However, for O/L out of 13 QTLs, 11 were major and stable with the highest PVE of 18.4%. All the major and stable QTLs for OLE, LIN, and O/L clustered on a 26.5 cM region (61.1–87.6 cM) on Ah19 (Figure 3, Table 1, and Supplementary Table 7). It was inferred that TMV 2 contributed to the decreased level of OLE, and increased level of LIN.

### Common QTL Clusters for the Productivity and Quality Traits

Three clusters were identified for the productivity and quality traits. Cluster 1 of 3.4 cM (46.7–50.1 cM on Ah02) was common for NPPP, PWPP, and SP. It showed the maximum PVE of 23.6, 20.9, and 12.6% for NPPP, PWPP, and SP, respectively (Figure 3 and Table 1). This region was highly stable for PWPP as it was detected over four seasons (S1, S2, S3, and S4). Also, the additive effects were high for this region, and the favorable alleles for NPPP, PWPP, and SP were contributed by TMV 2-NLM. Cluster 2 of 10.9 cM (7.1–8 cM on Ah16) carried the major QTL for PC and SP with a PVE of 7.9–13.3% and 11.9–19.9%, respectively (Figure 3 and Table 1). Cluster 3 of 26.5 cM (61.1–87.6 cM on Ah19) controlled OLE, LIN, and O/L with the PVE of 5.0–21.3%, 5.5–17.1%, and 6.0–18.4%, respectively, and this region was consistently stable over all the six seasons (Figure 3 and Table 1).

### Single Marker Analysis

Single marker analysis revealed that a total of six markers were significantly associated with OLE along with five each for LIN and O/L with PVE ≥ 10. Of them, four markers (Ah19_155127364, Ah19_155135344, Ah19_155135353, and Ah19_155172354) and one (Ah19_155165240) marker located, respectively, on Ah19 and Ah09 were common for OLE, LIN, and O/L. These markers were also identified to be the flanking markers by CIM. These associations were consistent over all the six seasons (Table 2). In addition, Ah10_36971572 located on Ah10 showed association with OLE only during the S4 season.

**Table 2:** Major SNP and AhTE markers identified using single marker analysis for the productivity and quality traits in the RIL population of TMV 2 and TMV 2-NLM of peanut.

**Trait**	**Marker**	**Chromosome**	**Position (cM)**	**LOD**	**PVE (%)**	**Season(s)**
OLE	Ah19_155127364	Ah19	67.1	8.4–12.3	9.2–13.1	S1, S2, S3, S4, S5, S6
	Ah19_155135344	Ah19	72.5	15.8–18.4	16.5–18.9	S1, S2, S3, S4, S5, S6
	Ah19_155135353	Ah19	72.6	16.7–18.7	15.6–19.2	S1, S2, S3, S4, S5, S6
	Ah19_155165240	Ah09	20.5	7.5–20.1	6.3–20.6	S1, S2, S3, S4, S5, S6
	Ah19_155172354	Ah19	77.6	7.9–20.8	6.8–21.2	S1, S2, S3, S4, S5, S6
	Ah10_36971572	Ah10	67.9	9.8	10.6	S4
LIN	Ah19_155127364	Ah19	67.1	8.2–12.6	8.9–13.5	S1, S2, S3, S4, S5, S6
	Ah19_155135344	Ah19	72.5	15.4–19.6	13.2–20.4	S1, S2, S3, S4, S5, S6
	Ah19_155135353	Ah19	72.6	12.3–20.3	13.1–20.7	S1, S2, S3, S4, S5, S6
	Ah19_155165240	Ah09	20.5	6.8–20.7	7.4–21.1	S1, S2, S3, S4, S5, S6
	Ah19_155172354	Ah19	77.6	7.7–21.5	8.5–21.8	S1, S2, S3, S4, S5, S6
O/L	Ah19_155127364	Ah19	67.1	8.3–11.1	9.1–11.9	S1, S2, S3, S4, S5, S6
	Ah19_155135344	Ah19	72.5	14.2–17	14.9–17.7	S1, S2, S3, S4, S5, S6
	Ah19_155135353	Ah19	72.6	14.7–17.3	15.5–18	S1, S2, S3, S4, S5, S6
	Ah19_155165240	Ah09	20.5	6–17.3	6.6–18	S1, S2, S3, S4, S5, S6
	Ah19_155172354	Ah19	77.6	6.9–18.3	7.6–18.9	S1, S2, S3, S4, S5, S6
PC	AhTE0281	Ah01	57.9	15	15.8	S1
	Ah03_127278448	Ah03	11.8	10.3	11.1	S1
	AhTE0087	Ah04	0.0	18.4	18.9	S1
	AhTE0275	Ah05	118.7	15.5	16.2	S1
	AhTE0120	Ah11	29.3	23	23.2	S1
	AhTE1110	Ah12	0.0	14.4	15.2	S1
	Ah12_118126407	Ah12	82.2	9.8–13.4	10.5–14.2	S1, S4
	AhTE0242	Ah16	0.0	9.6–21.6	10.4–21.9	S1, S4
	AhTE0060	Ah16	7.1	11.5–24.5	12.3–24.4	S1, S4
	AhTE1451	Ah18	74.6	11.1	11.9	S1
SP	AhTE0281	Ah01	57.9	18.8	19.3	S3
	AhTE0087	Ah04	0.0	10.4	11.2	S3
	AhTE0120	Ah11	29.3	17.5	18.1	S3
	AhTE0242	Ah16	0.0	13.1	13.9	S3

*LOD, Logarithm of Odds; PVE (%), Phenotypic coefficient of variation; LIN, Linoleic acid content; OLE, Oleic acid content; O/L, oleic to linoleic acid ratio; PC, Protein content and SP, Shelling percentage*.

A total of 10 markers were significantly associated with PC with a PVE of ≥10. Out of which AhTE0242 and AhTE0060 located at 0–7.16 cM were also identified by CIM. Three markers (Ah12_118126407, AhTE0242, and AhTE0060) showed association in S1 and S4 season, while seven markers (AhTE0281, Ah03_127278448, AhTE0087, AhTE0275, AhTE0120, AhTE1110, and AhTE1451) showed association only during the S1 season (Table 2). There were a few other markers associated with PC; however, they either showed relatively low PVE or appeared only during the specific seasons. SMA revealed that four markers (AhTE0281, AhTE0087, AhTE0120, and AhTE0242) were significantly associated with SP during the S3 season (Table 2). However, none of them was in the main effect QTL region detected for SP. For the remaining traits (OIL, NPPP, PWPP, and TW), none of the markers were detected as significant by SMA.

### Epistatic QTL Discovery for Productivity Traits

Epistatic QTL analysis for the complex productivity traits, namely, NPPP, PWPP, TW, and SP identified a total of 94 epiQTLs, such as 87 major epiQTLs. In total 9, 12, 14, and 52 major epiQTLs were identified for NPPP, PWPP, TW, and SP, respectively. It was found that four major main effect QTLs of NPPP and SP were involved in epistatic interaction with PVE more than 10% (Figure 3 and Table 3). The rest of the epiQTLs involved either the main effect QTLs with minor effects or new genomic regions (Figure 3 and Supplementary Table 8). Of the four epistatic interactions for NPPP involving the major and main effect QTLs, three emerged from a genomic region on Ah02 showing significant interaction with the regions on Ah04, Ah06, and Ah12 with the PVE of 15.8, 17.7, and 20.0%, respectively during S4 season. Also, the major QTL for NPPP on Ah04 showed epistatic interactions with its own proximal region (10 cM) during S2, S3, and S4 seasons with a maximum PVE of 28.5% (Figure 3 and Table 3). Out of the remaining five epiQTLs for NPPP, those on Ah06, Ah18, and Ah19 showed interactions with their own close proximal regions (20, 5, and 15 cM) with the highest PVE of 39.6, 13.6, and 35.8%, respectively. Furthermore, the epiQTLs on Ah03 and Ah10 showed significant interactions with genomic regions on Ah16 and Ah19 with maximum PVE of 13.4 and 11%, respectively for NPPP (Figure 3 and Supplementary Table 8).

**Table 3:** Major main effect QTLs showing major epistatic interaction for productivity traits across seasons in the RIL population of TMV 2 and TMV 2-NLM of peanut.

**Trait/QTL name**	**Locus 1**				**Locus 2**				**LOD**	**PVE (%)**	**Additive effect 1**	**Additive effect 2**	**AddbyAdd**	**Season(s)**
	**Chromosome 1**	**Position 1 (cM)**	**Left marker 1**	**Right marker 1**	**Chromosome 2**	**Position 2 (cM)**	**Left marker 2**	**Right marker 2**						
**NPPP**														
*qtlNPPP–Ah02-1*	Ah02	50	Ah02_100281747	Ah02_1558084	Ah04	10	AhTE0087	TC11H06	3.2	15.8	1.4	1.7	1.4	S4
*qtlNPPP–Ah02-2*	Ah02	50	Ah02_100281747	Ah02_1558084	Ah06	110	Ah16_110524270	AhTE2006	3.6	17.7	1.6	1.7	1.6	S4
*qtlNPPP–Ah02-3*	Ah02	50	Ah02_100281747	Ah02_1558084	Ah12	135	Ah12_1893158	Ah12_12348612	4.6	20	1.8	−1.8	−1.6	S4
*qtlNPPP–Ah04-1*	Ah04	0	AhTE0087	TC11H06	Ah04	5	AhTE0087	TC11H06	3.2–4.6	9.8–28.5	−2.4 to −1.3	1.6–2.7	−2.4 to −1.1	S4, S3, S2
**SP**														
*qtlSP–Ah03-6*	Ah03	65	Ah03_29890737	AhTE0178	Ah16	15	AhTE0060	Ah16_77480103	3.7–6.1	18.3–24.2	1.7–2.7	−1.8 to −1.8	2.3–3.1	S2, S4
*qtlSP–Ah08-5*	Ah08	15	Ah08_27217002	Ah17_20550255	Ah13	175	Ah13_80163117	Ah13_50074616	3.1–9.7	14–23.2	−4.7 to −3.6	−5 to −3.8	−5.5 to −4.2	S2, S4
*qtlSP–Ah12-1*	Ah12	95	Ah12_111595586	Ah12_40652945	Ah16	15	AhTE0060	Ah16_77480103	3.4–5.9	16.3–23.5	−4 to −3.6	−4.4 to −3.6	−4.7 to −3.5	S2, S1
*qtlSP–Ah13-2*	Ah13	165	Ah13_80163117	Ah13_50074616	Ah16	15	AhTE0060	Ah16_77480103	5.3–9.8	13.9–23.9	−4.4–13.5	−4.8–11.3	−5–12.1	S6,S2

*LOD, Logarithm of odds; PVE, Phenotypic variance explained; NPPP, Number of pods per plant; PWPP, Pod weight per plant (g); TW, Test weight (g); SP, Shelling percentage (%); S1, Rainy 2014; S2, Rainy 2015; S3, Rainy 2016; S4, Rainy 2017; S5, Rainy 2018; S6 and Post-rainy 2018*.

Of the four epistatic interactions for SP involving the major and main effect QTLs, a main effect QTL region on Ah16 (7.1–18 cM) showed epistatic interactions with Ah03, Ah12, and Ah13 with the maximum PVE of 24.2, 23.5, and 23.9%, respectively. Furthermore, a region (159.3–178.3 cM) on Ah13 with major main effect QTL also showed epistatic interactions with Ah08 recorded maximum PVE of 23.2% (Figure 3 and Table 3). In addition, a minor main effect QTL region on Ah01 for SP showed significant interaction with consecutive regions (at 60 and 80 cM) on Ah03 with the highest PVE of 22.3 and 22.6%. Among the remaining 47 epiQTLs, regions on Ah01, Ah05, Ah13, and Ah19 for SP were also involved in epistatic interaction with their own close proximal regions with PVE of 39.7, 30.4, 32.4, and 31.6%, respectively (Figure 3 and Supplementary Table 8).

For PWPP, none of the main-effect QTL was involved in epistatic interactions. However, the new epiQTL regions on Ah04, Ah05, Ah06, and Ah13 showed significant interactions with their own close proximal regions (5 cM) with the highest PVE of 36.4, 29.5, 43.3, and 26.7%, respectively. Apart from these, eight other epiQTLs appeared in at least two seasons with the major effect (PVE ≥10%) (Figure 3 and Supplementary Table 8).

Of the 14 epiQTLs detected for TW, a main effect minor QTL on Ah12 was involved in epistatic interaction with regions on Ah03, Ah07, and Ah14 with the highest PVE of 24.8, 28.5, and 15.8%, respectively. Among the remaining 11 epiQTLs, regions on Ah01, Ah03, Ah05, and Ah11 were also involved in epistatic interactions with maximum PVE of 17.6, 24.8, 28.4, and 14.3%, respectively. Most of these genomic regions identified in this study were important since they carried stable major QTL(s) which also showed significant epistatic interaction for various traits with PVE ≥ 10% across the seasons (Figure 3 and Supplementary Table 8).

### Putative Genes Identified in Major Main-Effect QTL Regions/Clusters

In total, the three clusters and four major and stable QTL regions were subjected to candidate gene discovery. In cluster 1, a 5 Mb region from the left flanking marker (Ah02_100281747) toward the common QTL peak for NPPP, PWPP, and SP was considered for gene discovery, and 360 genes were found (Table 4). In clusters 2 and 3, the region between the left and right flanking markers were considered, and 34 and 3 genes were found in the regions, respectively (Table 4).

**Table 4:** Putative genes identified in the major main effect QTL cluster/QTL regions for productivity and quality traits in the RIL population of TMV 2 and TMV 2-NLM of peanut.

**Region**	**QTL**	**Chromosome**	**Flanking markers**	**Region (cM)**	**Peak position (cM)**	**Region (Mb)**	**Genes**
**Cluster**							
1	*qNPPP–Ah02-1, qPWPP–Ah02-1* and *qSP–Ah02-1*	Ah02	Ah02_100281747–Ah02_1558084	46.7–50.1	48.7	5.0	360
2	*qPC–Ah16-2* and *qSP–Ah16-1*	Ah16	AhTE0060 (86223589) –Ah16_77480103	7.1–18.0	13.1	8.7	34
3^*^		Ah19	Ah19_155127299–Ah19_155179303	61.1–87.6	–	0.05	3
**QTL**							
1	*qSP–Ah13-1*	Ah13	Ah13_80163117–Ah13_50074616	159.3–178.3	163.3	30.0	249
2	*qPC–Ah16-1*	Ah16	AhTE0242 (3776007) –AhTE0060	0.0–7.1	3.0	5.0	259
3	*qOIL–Ah03-3*	Ah03	Ah03_142744376–AhTE1144	37.4–37.5	37.4	5.0	421
4	*qOIL–Ah05-1*	Ah05	Ah05_115061124–AhTE0470	108.2–115.3	111.2	5.0	333

* *Cluster 3 contains of 12, 13, and 11 major effect QTLs for OLE, LIN, and O/L, respectively*.

Of the four major QTL regions, a 5 Mb region from the left flanking marker to the QTL peak was employed for three; *qPC-Ah16-1, qOIL-Ah03-3*, and *qOIL-Ah05-1*, and 249, 421, and 333 genes were found in these regions, respectively (Table 4). The region between the two flanking markers was considered for *qSP-Ah13*-*1*, and 259 genes were found (Table 4). However, more studies are required to identify the candidate genes contributing to these traits.

Sixteen markers that were identified to be significantly associated with the traits by single marker analysis were checked for their location (genic and non-genic), effect, and probable function (Supplementary Table 9). Of them, 10 were found to be located in the intergenic regions and two each were located in the exonic, 5′ UTR, and intronic regions (Supplementary Table 9). AhTE0281 being located in the 16th exonic region of *Arahy.7A57YA* on Ah16 contributed for SP and PC (Supplementary Table 9). In addition, Ah12_118126407 being located in the second exon of *Arahy.J5SZ1I* (Ah12) governed PC. AhTE1451 being located in the 5′ UTR of *Arahy.CH9B83* (Ah18) also governed PC (Supplementary Table 9). Similarly, an SNP at 155172354 bp being located in the 5′ UTR of the gene *Arahy.X7PJ8H* on Ah19 contributed for OLE, LIN, and O/L (Supplementary Table 9). Also, both Ah19_155135344 and Ah19_155135353 being located in the 11th intron of *Arahy.MZJT69* on Ah19 contributed for OLE, LIN, and O/L (Supplementary Table 9).

### Confirmation of the QTLs and Markers

The two stable major QTLs *qPC-Ah16-1* and *qOIL-Ah03-3* were selected for validation using the other eight genotypes (Supplementary Table 10). The closest flanking markers; AhTE0242 for *qPC-Ah16-1* and AhTE1144 for *qOIL-Ah03-3* were used for genotyping. The *t*-test was significant (*p* < 0.05) for AhTE0242 and AhTE1144 markers, indicating a strong validation of the markers and thereby the QTL for PC and OIL (Table 5).

**Table 5:** Validation of QTL and markers linked to oil and protein content in peanut.

**QTL**	**Closest marker**	***t*-value**	***p*-value^a^**	**LOD**	**PVE (%)**
*qPC–Ah16-1*	AhTE0242	2.13	0.011^S^	0.8	37.0
*qOIL–Ah03-3*	AhTE1144	1.94	0.014^S^	0.33	17.0

a *Student's t-test (p < 0.05) was performed to identify co-segregation between distinct allele and phenotype; S, Significant; NS, Non-significant*.

*Phenotypic values and allelic pattern of genotypes considered for QTL validation given in Supplementary Table 9*.

## Discussion

In our previous study, the RIL population derived from TMV 2 and TMV 2-NLM was used for constructing the AhTE marker-based genetic map and identifying the QTL for important taxonomic and productivity traits (Hake et al., 2017). Since the parents and the RILs also differed for quality traits, an effort was made in the present study to map the quality traits using an improved genetic map with extensive multi-season phenotypic data on the productivity and quality traits collected over six seasons and GBS-derived SNP data. In this population, GBS could identify more number of SNPs (713) polymorphic between TMV 2 and TMV 2-NLM than the number of SNPs (31 SNP loci) detected using the ddRAD-Seq in the previous study (Hake et al., 2017). This could be due to the differences in the methodology, especially the use of a four-base cutter and a six-base cutter in ddRAD-Seq, while only a four-base cutter in GBS for generating the DNA fragments. With the 865 markers available for mapping, a total of 700 markers loci were mapped on the genetic map of 2,438.1 cM. The map density was increased to 3.5 cM/loci as compared with a previous genetic map where a total of 91 marker loci were mapped onto a genetic map of 1,205.6 cM with 18.1 cM/loci map density (Hake et al., 2017). In the previous genetic mapping studies with GBS or WGRS (whole genome re-sequencing) or SNP array, the diploid reference genomes of *Arachis duranensis* and *Arachis ipaensis* were used for SNP calling (Dodia et al., 2019; Gangurde et al., 2020). However, the present study used the tetraploid peanut (*A. hypogaea*) genome (Bertioli et al., 2019) as the reference for the true representative SNP calling.

The genomic regions controlling NPPP, PWPP, SP, TW, PC, OIL, OLE, LIN, and the OLE to LIN ratio (O/L) were identified using the phenotypic data generated over six seasons and the newly constructed improved genetic map with SNP, AhTE, and SSR markers. In this study, the main effect QTLs with major contributions (>10% PVE) were detected for all the traits except for TW. Likewise, the genomic region showing epistatic interactions for productivity traits (NPPP, PWPP, SP, and TW) were also identified. It was noticed that the traits identified with the major QTL showed higher GCV and broad sense heritability. The QTLs for highly correlated traits, such as OLE, LIN, and O/L shared a common marker interval on chromosome Ah19. Similarly, QTLs for NPPP, PWPP, and SP shared common marker interval QTLs on Ah02 and for PC and SP on chromosome Ah16. Though G × E interactions were significant for all the traits, stable QTL regions could be detected for the majority of the traits in this study. Based on the stability of QTLs across the seasons, we identified the QTL clusters and markers for validation and subsequent deployment in molecular breeding for improving the traits. The QTL region flanked by Ah02_100281747-Ah02_1558084 on chromosome Ah02, either through its main effect or epistatic interactions, showed significant contribution for NPPP (through *qNPPP-Ah02*-*1*), PWPP (through *qPWPP-Ah02*-*1*), and SP (through *qSP-Ah02*-*1*). The QTL regions on Ah06 and Ah19 were also important for NPPP. A QTL on Ah06 was important for PWPP only through its epistatic interaction. QTLs on chromosomes Ah13 and Ah16 for SP showed main as well as epistatic effects, while the same and its consecutive region on Ah16 also showed main QTL for PC. Therefore, selection based on the QTL region at 7.1–18.0 cM on chromosome Ah16 might improve not only SP but also PC. This was also supported by the significant positive correlation between SP and PC that was observed in this study and the previous study (Kumar et al., 2014). Moreover, SMA also showed that four markers contributed to both SP and PC. Furthermore, validation of these markers across the seasons and genotypes might indicate their utility in the marker-assisted breeding for simultaneous improvement of PC and SP since seasonal variation for PC has been reported earlier (Sarvamangala et al., 2011). The main effect QTLs on Ah12 for TW also showed epistatic interactions with genomic regions on Ah05 and Ah14. This might help in transferring the main effect and epiQTLs simultaneously to improve kernel weight. The selection based on the main effects of the QTLs on chromosome Ah03 (*qOIL-Ah03-3*) and Ah05 (*qOIL-Ah05-1*) could advance the genetic gains for OIL. Similarly, the main effect of the QTL clusters at 61.1–87.6 cM on chromosome Ah19 could contribute to improving O/L (increased OLE and decreased LIN). Single marker analysis also showed the significant association of five markers from this region with OLE, LIN, and O/L stably across the seasons. The QTL regions in the close vicinity on a few chromosomes (Ah01, Ah04, Ah05, Ah06, Ah13, and Ah19) showing epistatic interaction for the productivity traits might be resolved by fine mapping so that the selection becomes more effective. Parent TMV 2-NLM could be considered as the source of favorable allele at the region on Ah02 which contributed for NPPP, PWPP, SP, and TW. In addition, the favorable allele from TMV 2 at 19 cM region on Ah13 might be considered while selecting for SP.

Two of the QTL regions identified in this study were validated using other genotypes. A region 7.1–18.0 cM on chromosome Ah16 for PC and 37.4–37.5 cM region on chromosome Ah03 for OIL showed strong validation, indicating that these QTLs are genotype-independent. Many QTLs were also consistent as they were reported to be co-localized in the previous studies thereby supporting their utility. The region at 46.7–50.1 cM on chromosome Ah02 identified for NPPP, PWPP, SP, and TW in this study was previously detected for pod length (Fonceka et al., 2012), seed length (Zhang et al., 2019), and SP (Chavarro et al., 2020). Similarly, the 37.4–37.5 cM region on chromosome Ah03 linked to OIL was reported by Sarvamangala et al. (2011). The single region on Ah19 linked to OLE, LIN, and O/L was consistent with the study of Pandey et al. (2014) and Shasidhar et al. (2017). However, a stable QTL region (159.3–178.3 cM) on Ah13 reported in this study for SP with the LOD score of 3.2–35.5 and PVE of 16.3–52.8% over four seasons differed from the region (60.3–64.7 cM) reported by Zhang et al. (2019) for seed length.

With the availability of the genome sequence for the diploid ancestors and the cultivated peanut now (Bertioli et al., 2019), candidate gene discovery is relatively easy as it has been reported for SP (Luo et al., 2017), seed weight (Gangurde et al., 2020), TW (Wang et al., 2019), stem rot resistance (Dodia et al., 2019), and foliar disease resistance (Shirasawa et al., 2018). Here, putative gene discovery was performed in the three QTL clusters and four major QTL regions; 3.4 cM region on Ah02 chromosome identified for NPPP, PWPP, SP, and TW, 10.9 cM region on Ah16 for SP and PC, and 26.5 cM region on Ah19 for OLE, LIN, and O/L. There were 360 predicted genes in the 3.4 cM region on Ah02, while 34 genes were identified in the 10.9 cM region on Ah16. The 26.5 cM QTL cluster on Ah19 had only three predicted genes. Furthermore, this region was in the vicinity of *FAD2B* gene that determines OLE and LIN content and therefore used widely for marker-assisted breeding (Jadhav et al., 2021).

Putative gene discovery was also performed in the four stable major QTL regions, such as *qSP-Ah13*-*1* at 19 cM on Ah13 for SP, *qSP-Ah16*-*1* at 7.1 cM on Ah16 for PC, and QTLs (*qOIL_Ah03-3* at 0.1 cM on Ah03 and *qOIL_Ah05-1* at 7.1 cM on Ah05) for OIL. Since the flanking markers for these QTLs were distanced quite apart, the markers close to the peak were selected, and a physical distance of 5 Mb toward the QTL peak was searched for the predicted genes. This effort identified a large number of genes (249–421) across the four QTL regions. Therefore, it may be too primitive to conclude about the candidate genes for the traits observed in this study, and fine mapping might be essential to resolve the regions and identify the candidate genes for effective use in marker-assisted breeding.

Also, the marker loci associated with the traits identified through SMA were further considered for identifying the candidate genes. In total, five candidate genes could be identified; they included *Arahy.7A57YA* (coding for *ARM repeat superfamily protein*) for SP and PC, *Arahy.J5SZ1I* (coding for *syntaxin of plants*) and *Arahy.CH9B83* (coding for *phosphatidylinositol 3,4,5-trisphosphate 3-phosphatase and dual-specificity protein phosphatase PTEN-like isoform*) for both SP and PC, and *Arahy.MZJT69* and *Arahy.X7PJ8H* for OLE, LIN, and O/L. *Arahy.MZJT69* (*coding for receptor-like protein kinase 4*) and *Arahy.X7PJ8H* (coding for p*rotein kinase superfamily*) altered the phenotype probably through SNP, while *Arahy.7A57YA* (*coding for ARM repeat superfamily protein*) and *Arahy.CH9B83* (*coding for phosphatidylinositol 3,4,5-trisphosphate 3-phosphatase and dual-specificity protein phosphatase PTEN-like isoform*) genes contributed to the phenotype probably through the transpositional activity of *AhMITE1* as reported earlier with AhTE0391 marker in *Aradu.7N61X* (*coding for alpha-glucosidase*) (Hake et al., 2017).

Overall, this study contributed to the development of an improved map with 700 markers for a unique mapping population derived from an elite variety TMV 2 and its mutant, which probably offers a greater opportunity for subtracting a major portion of the genome common to both the parents and considering probably a small portion of the genome that differs between the parents for mapping the traits. This fact was pronounced both in this study as well as the previous study (Hake et al., 2017), which together reported the mapping of taxonomical, productivity, and quality traits in peanut.

## Data Availability Statement

The datasets presented in this study can be found in online repositories. The names of the repository/repositories and accession number(s) can be found at: BioProject: PRJNA744708.

## Author Contributions

MJ conducted phenotyping, genotyping with AhTE and SSR markers, analyzed the data, and contributed to manuscript draft preparation. AY and SM assisted in generating the phenotypic and genotypic data. SG analyzed data and contributed to manuscript preparation. AH collected the data on phenotyping in two seasons and generated the AhTE marker data. SP and MG developed the mapping population and contributed to planning the study. KS performed SNP effect analysis. RV contributed for generating GBS data. MP contributed to generating GBS data, guiding genetic analysis, and planning manuscript content. RB conceptualized the study, planned manuscript content, coordinated with co-authors, and finalized the manuscript. All authors contributed to the article and approved the submitted version.

## Funding

The funding support for genotyping-by-sequencing (GBS) performed in this study was received from National Agricultural Science Fund (NASF) of Indian Council of Agricultural Research, India.

## Conflict of Interest

The authors declare that the research was conducted in the absence of any commercial or financial relationships that could be construed as a potential conflict of interest.

## Publisher's Note

All claims expressed in this article are solely those of the authors and do not necessarily represent those of their affiliated organizations, or those of the publisher, the editors and the reviewers. Any product that may be evaluated in this article, or claim that may be made by its manufacturer, is not guaranteed or endorsed by the publisher.

## References

[B1] Arya S. S. Salve A. R. Chauhan S. (2016). Peanuts as functional food: a review. J. Food Sci. Technol. 53, 31–41. 10.1007/s13197-015-2007-9 26787930 PMC4711439

[B2] Bertioli D. J. Cannon S. B. Froenicke L. Huang G. Farmer A. D. Cannon E. K.. (2016). The genome sequences of *Arachis duranensis* and *Arachis ipaensis*, the diploid ancestors of cultivated peanut. Nat. Genet. 48, 438–446. 10.1038/ng.3517 26901068

[B3] Bertioli D. J. Jenkins J. Clevenger J. Dudchenko O. Gao D. Seijo G.. (2019). The genome sequence of segmental allotetraploid peanut *Arachis hypogaea*. Nat. Genet. 51, 877–884. 10.1038/s41588-019-0405-z 31043755

[B4] Bradbury P. J. Zhang Z. Kroon D. E. Casstevens T. M. Ramdoss Y. Buckler E. S. (2007). TASSEL: software for association mapping of complex traits in diverse samples. Bioinformatics 23, 2633–2635. 10.1093/bioinformatics/btm308 17586829

[B5] Browning B. L. Zhou Y. Browning S. R. (2018). A one-penny imputed genome from next-generation reference panels. Am. J. Hum. Genet. 103, 338–348. 10.1016/j.ajhg.2018.07.015 30100085 PMC6128308

[B6] Chavarro C. Chu Y. Holbrook C. Isleib T. Bertioli D. Hovav R.. (2020). Pod and seed trait QTL identification to assist breeding for peanut market preferences. G3 Genes Genomes Genet. 10, 2297–2315. 10.1534/g3.120.401147 32398236 PMC7341151

[B7] Chen X. Li H. Pandey M. K. Yang Q. Wang X. Garg V.. (2016). Draft genome of the peanut A-genome progenitor (*Arachis duranensis*) provides insights into geocarpy, oil biosynthesis, and allergens. Proc. Natl. Acad. Sci. U.S.A. 113, 6785–6790. 10.1073/pnas.1600899113 27247390 PMC4914189

[B8] Chen X. Lu Q. Liu H. Zhang J. Hong Y. Lan H.. (2019). Sequencing of cultivated peanut, *Arachis hypogaea*, yields insights into genome evolution and oil improvement. Mol. Plant 12, 920–934. 10.1016/j.molp.2019.03.005 30902685

[B9] Cuc L. M. Mace E. S. Crouch J. H. Quang V. D. Long T. D. Varshney R. K. (2008). Isolation and characterization of novel microsatellite markers and their application for diversity assessment in cultivated groundnut (*Arachis hypogaea*). BMC Plant Biol. 8:55. 10.1186/1471-2229-8-55 18482440 PMC2416452

[B10] Dodia S. M. Joshi B. Gangurde S. S. Thirumalaisamy P. P. Mishra G. P. Narandrakumar D.. (2019). Genotyping-by-sequencing based genetic mapping reveals large number of epistatic interactions for stem rot resistance in groundnut. Theor. Appl. Genet. 132, 1001–1016. 10.1007/s00122-018-3255-7 30539317

[B11] Fonceka D. Tossim H. A. Rivallan R. Vignes H. Faye I. Ndoye O.. (2012). Fostered and left behind alleles in peanut: interspecific QTL mapping reveals footprints of domestication and useful natural variation for breeding. BMC Plant Biol. 12:26. 10.1186/1471-2229-12-26 22340522 PMC3312858

[B12] Gangurde S. S. Wang H. Yaduru S. Pandey M. K. Fountain J. C. Chu Y.. (2020). Nested-association mapping (NAM)-based genetic dissection uncovers candidate genes for seed and pod weights in peanut (*Arachis hypogaea*). Plant Biotechnol. J. 18, 1457–1471. 10.1111/pbi.13311 31808273 PMC7206994

[B13] Gayathri M. Shirasawa K. Varshney R. K. Pandey M. K. Bhat R. S. (2018). Development of new *AhMITE1* markers through genome-wide analysis in peanut (*Arachis hypogaea* L.). BMC Res. Notes 11:10. 10.1186/s13104-017-3121-8 29310707 PMC5759262

[B14] Hake A. A. Shirasawa K. Yadawad A. Sukruth M. Patil M. Nayak S. N.. (2017). Mapping of important taxonomic and productivity traits using genic and non-genic transposable element markers in peanut (*Arachis hypogaea* L.). PLoS ONE 12:e0186113. 10.1371/journal.pone.0186113 29040293 PMC5645101

[B15] Han S. Yuan M. Clevenger J. P. Li C. Hagan A. Zhang X.. (2018). A SNP-based linkage map revealed qtls for resistance to early and late leaf spot diseases in peanut (*Arachis hypogaea* L.). Front. Plant Sci. 9:1012. 10.3389/fpls.2018.01012 30042783 PMC6048419

[B16] Jadhav M. P. Patil M. D. Hampannavar M. Venkatesh, Pavana D. Shirasawa K. Pasupuleti J.. (2021). Enhancing oleic acid content in two commercially released peanut varieties through marker-assisted backcross breeding. Crop Sci. 61, 2435–2443. 10.1002/csc2.20512

[B17] Kolekar R. M. Sujay V. Shirasawa K. Sukruth M. Khedikar Y. P. Gowda M. V. C.. (2016). QTL mapping for late leaf spot and rust resistance using an improved genetic map and extensive phenotypic data on a recombinant inbred line population in peanut (*Arachis hypogaea* L.). Euphytica 209, 147–156. 10.1007/s10681-016-1651-0

[B18] Kosambi D. D. (1943). The estimation of map distances from recombination values. Ann. Eugen. 12, 172–175. 10.1111/j.1469-1809.1943.tb02321.x

[B19] Kumar C. P. Rekha R. Venkateswarulu O. Vasanthi R. P. (2014). Correlation and path coefficient analysis in groundnut (*Arachis hypogaea L*.). Int. J. Appl. 5, 8–11. Available online at: https://www.semanticscholar.org/paper/Correlation-and-Path-Coefficient-Analysis-in-L.).-Kumar-Rekha/8e0f89bdc5831451d1b5522b82e4381b2c72c73b

[B20] Li H. Durbin R. (2009). Fast and accurate short read alignment with Burrows–Wheeler transform. Bioinformatics 25, 1754–1760. 10.1093/bioinformatics/btp324 19451168 PMC2705234

[B21] Lu Q. Li H. Hong Y. Zhang G. Wen S. Li X.. (2018). Genome sequencing and analysis of the peanut B-genome progenitor (*Arachis ipaensis*). Front. Plant Sci. 9:604. 10.3389/fpls.2018.00604 29774047 PMC5943715

[B22] Luo H. Guo J. Yu B. Chen W. Zhang H. Zhou X.. (2021). Construction of ddRADseq-based high-density genetic map and identification of quantitative trait loci for trans-resveratrol content in peanut seeds. Front. Plant Sci. 12, 438. 10.3389/fpls.2021.644402 33868342 PMC8044979

[B23] Luo H. Xu Z. Li Z. Li X. Lv J. Ren X.. (2017). Development of SSR markers and identification of major quantitative trait loci controlling shelling percentage in cultivated peanut (*Arachis hypogaea* L.). Theor. Appl. Genet. 130, 1–14. 10.1007/s00122-017-2915-3 28508097 PMC5511596

[B24] Pandey M. K. Agarwal G. Kale S. M. Clevenger J. Nayak S. N. Sriswathi M.. (2017). Development and evaluation of a high density genotyping ‘Axiom_Arachis’ array with 58 K SNPs for accelerating genetics and breeding in groundnut. Sci. Rep. 7:40577. 10.1038/srep40577 28091575 PMC5238394

[B25] Pandey M. K. Gautami B. Jayakumar T. Sriswathi M. Upadhyaya H. D. Gowda M. V. C.. (2012). Highly informative genic and genomic SSR markers to facilitate molecular breeding in cultivated groundnut (*Arachis hypogaea*). Plant Breed. 131, 139–147. 10.1111/j.1439-0523.2011.01911.x

[B26] Pandey M. K. Pandey A. K. Kumar R. Nwosu C. V. Guo B. Wright G. C.. (2020). Translational genomics for achieving higher genetic gains in post-genome era in groundnut. Theor. Appl. Genet. 133, 1679–1702. 10.1007/s00122-020-03592-2 32328677 PMC7214508

[B27] Pandey M. K. Wang M. L. Qiao L. Feng S. Khera P. Wang H.. (2014). Identification of QTLs associated with oil content and mapping *FAD2* genes and their relative contribution to oil quality in peanut (*Arachis hypogaea* L.). BMC Genet. 15:133. 10.1186/s12863-014-0133-4 25491595 PMC4278341

[B28] Pattanashetti S. K. (2005). Genetic analysis of mutational origin of diversity in groundnut (Arachis hypogaea L.) (Ph. D.). University of Agricultural Sciences, Dharwad, India.

[B29] Prasad M. V. R. Kaul S. Jain H. K. (1984). Induced mutants of peanut (*Arachis hypogaea* L.) for canopy and pod bearing characters. Indian J. Genet. Plant Breed. 44, 25–34.

[B30] Rathnakumar A. L. Singh R. Parmar D. L. Mishra J.B. (2013). Groundnut a Crop Profile and Compendium of Notified Varieties of India. Junagadh: Directorate of Groundnut Rserach (ICAR).

[B31] Sarvamangala C. Gowda M. V. C. Varshney R. K. (2011). Identification of quantitative trait loci for protein content, oil content and oil quality for groundnut (*Arachis hypogaea* L.). Field Crops Res. 122, 49–59. 10.1016/j.fcr.2011.02.010

[B32] Sharma V. P. (2017). Oilseed Production in India the Problems and Prospects. Ahmedabad: Springer.

[B33] Shasidhar Y. Vishwakarma M. K. Pandey M. K. Janila P. Variath M. T. Manohar S. S.. (2017). Molecular mapping of oil content and fatty acids using dense genetic maps in groundnut (*Arachis hypogaea* L.). Front. Plant Sci. 8:794. 10.3389/fpls.2017.00794 28588591 PMC5438992

[B34] Shirasawa K. Bhat R. S. Khedikar Y. P. Sujay V. Kolekar R. M. Yeri S. B.. (2018). Sequencing analysis of genetic loci for resistance for late leaf spot and rust in peanut (*Arachis hypogaea* L.). Front. Plant Sci. 9:1727. 10.3389/fpls.2018.01727 30534132 PMC6275244

[B35] Van Ooijen J. (2006). JoinMap® 4, Software for the Calculation of Genetic Linkage Maps in Experimental Populations. Wageningen: Kyazma BV. p. 33.

[B36] Voorrips R. (2002). MapChart: software for the graphical presentation of linkage maps and QTLs. J. Heredity 93, 77–78. 10.1093/jhered/93.1.77 12011185

[B37] Wang J. Li H. Zhang L. Meng L. (2014). Users' Manual of QTL IciMapping. The Quantitative Genetics Group, Institute of Crop Science, Chinese Academy of Agricultural Sciences (CAAS), Beijing; Genetic Resources Program, International Maize and Wheat Improvement Center (CIMMYT), Mexico.

[B38] Wang J. Yan C. Li Y. Li C. Zhao X. Yuan C.. (2019). GWAS discovery of candidate genes for yield-related traits in peanut and support from earlier QTL mapping studies. Genes 10:803. 10.3390/genes10100803 31614874 PMC6826990

[B39] Wang J. Yan C. Shi D. Zhao X. Yuan C. Sun Q.. (2021). The genetic base for peanut height-related traits revealed by a meta-analysis. Plants 10:1058. 10.3390/plants10061058 34070508 PMC8227209

[B40] Wang S. Basten C. Zeng Z. (2007). Windows QTL cartographer 2.5. North Carolina State University.

[B41] Yin D. Ji C. Ma X. Li H. Zhang W. Li S.. (2018). Genome of an allotetraploid wild peanut *Arachis monticola*: a *de novo* assembly. GigaScience 7:giy066. 10.1093/gigascience/giy066 29931126 PMC6009596

[B42] Zeng Z. B. (1994). Precision mapping of quantitative trait loci. Genetics 136, 1457–1468. 10.1093/genetics/136.4.1457 8013918 PMC1205924

[B43] Zhang S. Hu X. Miao H. Chu Y. Cui F. Yang W.. (2019). QTL identification for seed weight and size based on a high-density SLAF-seq genetic map in peanut (*Arachis hypogaea* L.). BMC Plant Biol. 19:537. 10.1186/s12870-019-2164-5 31795931 PMC6892246

[B44] Zhao Y. Zhang C. Chen H. Yuan M. Nipper R. Prakash C.. (2016). QTL mapping for bacterial wilt resistance in peanut (*Arachis hypogaea* L.). Mol. Breed. 36, 1–11. 10.1007/s11032-015-0432-0 26869849 PMC4735223

[B45] Zhou X. Guo J. Pandey M. K. Varshney R. K. Huang L. Luo H.. (2021). Dissection of the genetic basis of yield-related traits in the chinese peanut mini-core collection through genome-wide association studies. Front. Plant Sci. 12:664. 10.3389/fpls.2021.637284 34093605 PMC8174301

[B46] Zhuang W. Chen H. Yang M. Wang J. Pandey M. K. Zhang C.. (2019). The genome of cultivated peanut provides insight into legume karyotypes, polyploid evolution and crop domestication. Nat. Genet. 51, 865–876. 10.1038/s41588-019-0402-2 31043757 PMC7188672

