# Supplementary figures and images for: Genotyping-by-Sequencing Based Genetic Mapping Identified Major and Consistent Genomic Regions for Productivity and Quality Traits in Peanut
Source: Front Plant Sci. 2021 Sep 23;12:668020. doi: 10.3389/fpls.2021.668020 (PMC8495222; doi:10.3389/fpls.2021.668020)

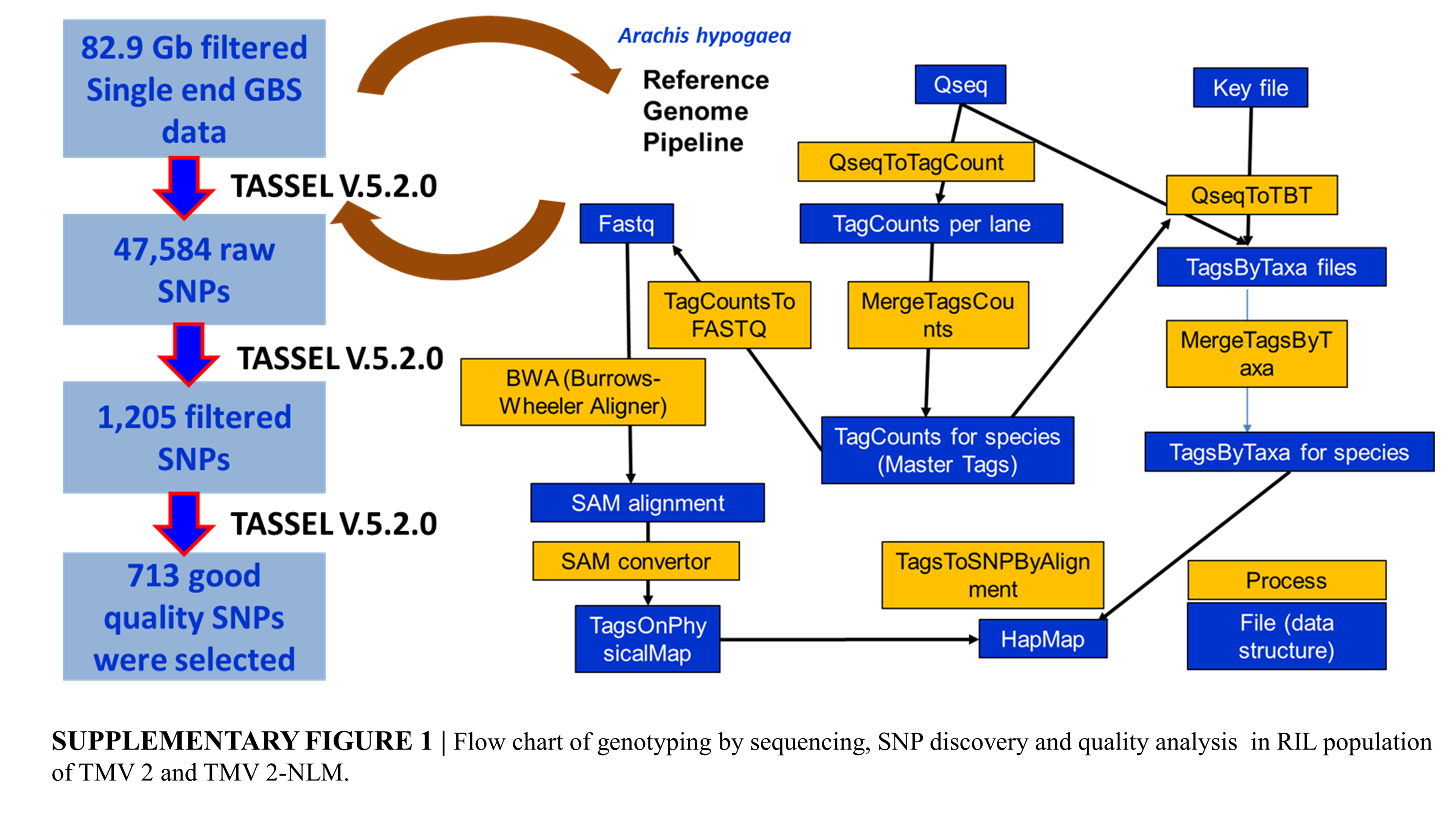

Supplement: Supplementary file 11 [file Image_1.TIF]

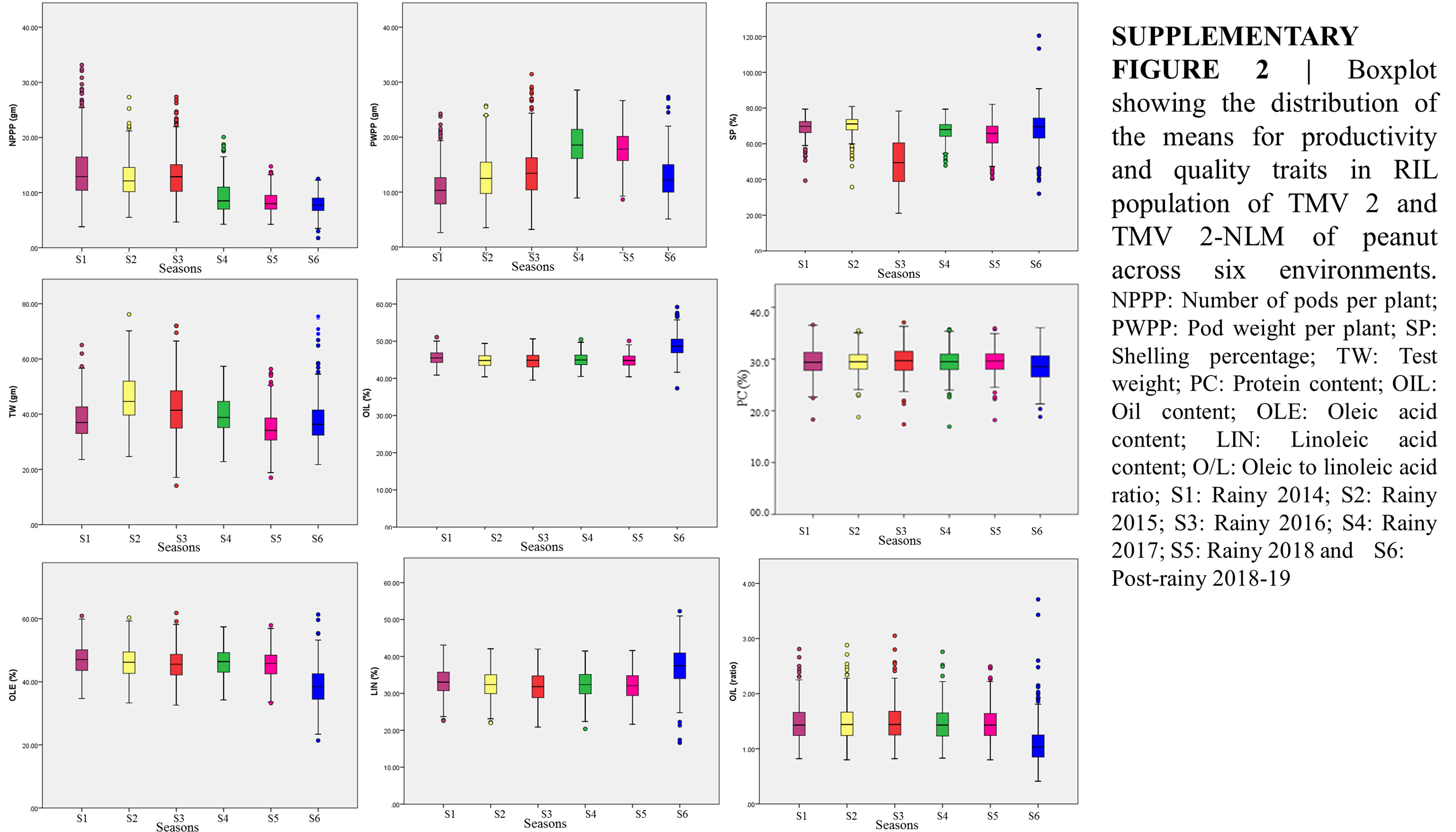

Supplement: Supplementary file 12 [file Image_2.TIF]
